# Tailoring centripetal metamaterial with superelasticity and negative Poisson’s ratio for organic solvents adsorption

**DOI:** 10.1126/sciadv.abo1014

**Published:** 2022-09-30

**Authors:** Li Tian, Jinshan Yang, Xiao You, Mengmeng Wang, Xiaoyin Ren, Xiangyu Zhang, Shaoming Dong

**Affiliations:** ^1^ State Key Laboratory of High Performance Ceramics and Superfine Microstructure, Shanghai Institute of Ceramics, Chinese Academy of Sciences, Shanghai 200050, China.; ^2^ Structural Ceramics and Composites Engineering Research Center, Shanghai Institute of Ceramics, Chinese Academy of Sciences, Shanghai 200050, China.; ^3^ University of Chinese Academy of Sciences, Beijing 100039, China.; ^4^ Center of Materials Science and Optoelectronics Engineering, University of Chinese Academy of Sciences, Beijing 100049, China.

## Abstract

Graphene metamaterials with a radial-like structure and negative Poisson’s ratio (NPR) were assembled using a unique centripetal freezing technique. Driven by the centripetal temperature gradient, ice crystals were grown toward the center of an aqueous graphene dispersion and form a radially arranged skeleton. A reentrant structure was formed at the diagonal of the monolith as the ice crystals sublimate. The obtained centripetal graphene metamaterial (CGM) was endowed with NPR response. CGM maintained NPR under 50% compression, which reached a minimum (−0.18) at 10% strain. After 50 compressive cycles at 50% strain, CGM retained approximately 96% of the original compressive strength. The radial channels endowed CGM with fast absorption kinetics, and the NPR response effectively accommodated the damage caused by volume shrinkage during repeated adsorption-regeneration cycles. This strategy is an effective method for achieving NPR response and improving the mechanical properties of porous materials.

## Teaser

Centripetal freeze-casting is applied to construct graphene metamaterial with reentrant structure and auxetic behavior.

## INTRODUCTION

Porous materials, such as aerogels (*1*), sponges (*2*), foams (*3*), and microlattices (*4*, *5*), have been widely used in thermal insulation (*6*, *7*), environmental protection (*8*), and so on. These applications require materials to have excellent mechanical properties and functionality under the conditions of as lightweight as possible, which is mutually exclusive but feasible. As one of the porous materials, graphene aerogel (GA) has high porosity, interconnected channels among other attributes (*9*–*11*). So far, a variety of synthetic methods have been used for the preparation of GA, such as chemical vapor deposition, hydrothermal synthesis, three-dimensional (3D) printing, etc. (*12*–*15*), but these methods often require complex synthesis processes, high costs, or involve toxic reagents. As a green and simple assembly method, freeze-casting is often used to fabricate GAs with tailored porous structure (*16*–*18*), which offers great technological promise for organic solvent absorption and other applications (*19*–*21*). However, few studies have focused on metamaterial field and application prospects, which are intimately bound up with ordered porous structure (*22*). Among them, negative Poisson’s ratio (NPR) metamaterial exhibits negative index property, which has a substantial enhancement on the mechanical properties and effectively deal with the damage caused by volume shrinkage (*23*, *24*).

Poisson’s ratio (ν = −ε*_x_*/ε*_z_*) is usually defined as the negative ratio of the transverse strain (ε*_x_*) to the longitudinal strain (ε*_z_*) in the elastic loading directions, which characterizes transverse deformation characteristics under deformation (*25*, *26*). Contrary to positive Poisson’s ratio materials, materials with NPR exhibit lateral expansion (contraction) when stretched (compressed). Moreover, the density of the material increases rapidly when the material is subjected to uniaxial load and forms reentrant configurations. These metamaterials cause the material to shrink in all directions and imply superior properties [e.g., higher shear modulus, indentation resistance, and energy absorption (*27*–*30*)]. Topological structure design and simulation also further prove the superiority of NPR structure (*31*–*33*). Briefly, the implementation of the NPR effect depends on the structural design rather than compositions. However, most reports are limited to the 2D NPR structure, 3D configuration combined with optimized NPR structural design has rarely been discussed, and the response mechanism of NPR and potential applications need to be further explored.

In this study, graphene metamaterials with centripetal reentrant structures and NPRs are designed and synthesized by centripetal freeze-casting. In this method, a temperature gradient is generated from the inner surface of the copper mold to the center, and finally, the graphene oxide (GO) sheets assemble into a centripetal aligned structure during growth of the ice crystals. Centripetal freeze-casting confers a unique NPR effect to the centripetal graphene metamaterials (CGMs), wherein the materials consistently show an NPR when compressed, reaching −0.18 at 10% strain. CGM is highly efficient for the absorption of organic solvents, with a chloroform adsorption capacity of up to 358 g·g^−1^, and exhibits good recyclability. The mechanism underlying the NPR effect is analyzed by observing the microstructural evolution.

## RESULTS AND DISCUSSION

### Synthesis mechanism of CGM

**Fig. 1.**
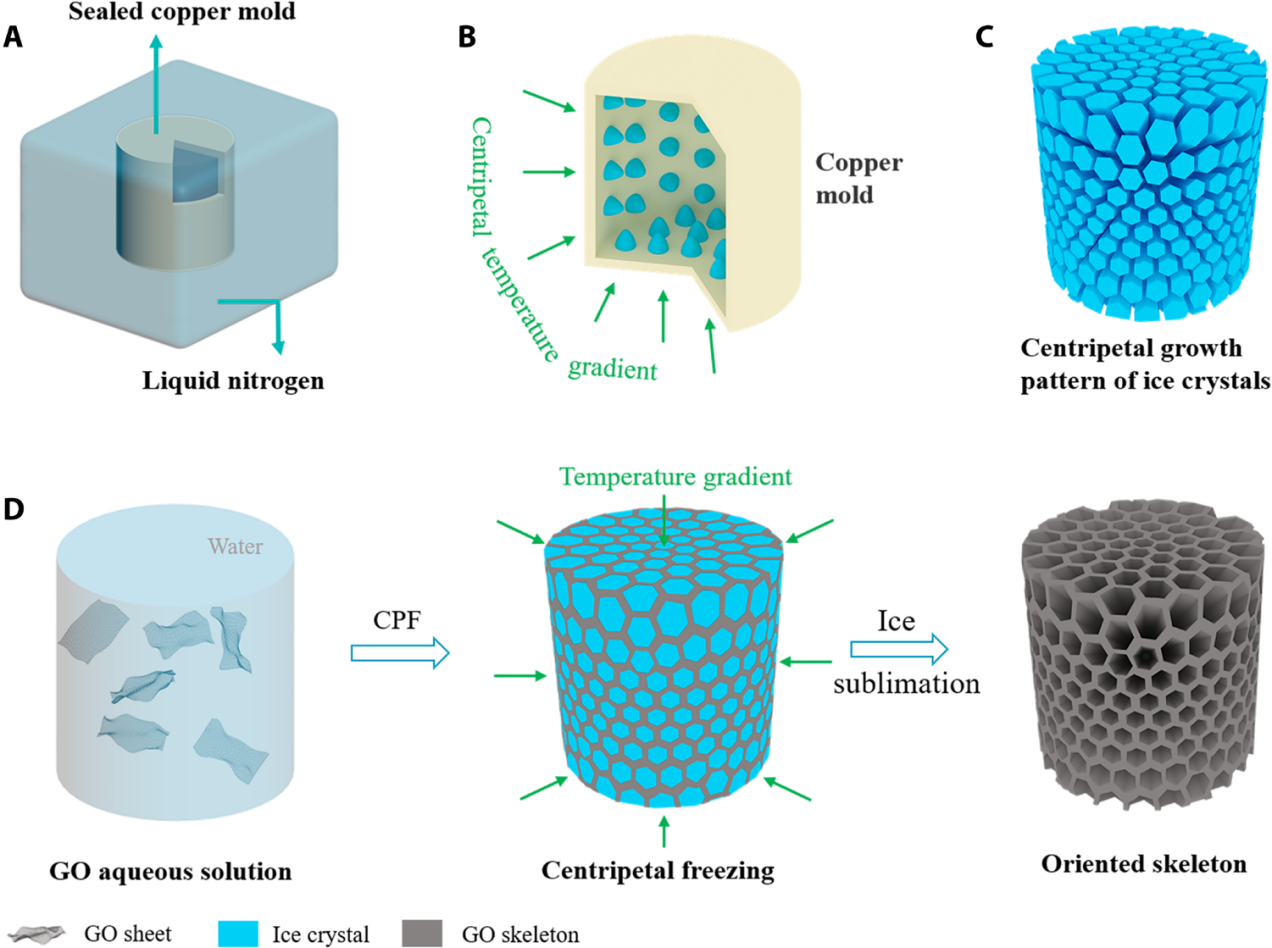
Illustration of a centripetal graphene metamaterial fabricated by centripetal freezing. (**A**) Sealed copper mold is used as a freezing device to produce an orderly structure with its inside filled with the solution; the black part represents the GO solution. (**B**) Centripetal temperature gradient induced by the sealed copper mold. (**C**) Model of the ice crystals growing along radial directions. (**D**) Scheme of the fabrication process.

The synthesis process involves complex and dynamic interactions between ice crystals and blocks (*34*). By controlling the freezing conditions, the obtained microstructure can be effectively designed. So far, the main freeze-casting methods are conventional strategies that contain “common freezing (CF)” and “unidirectional freezing (UF)” (*35*, *36*). CF usually involves putting a container of liquid into a refrigerator to form a random structure (*37*, *38*). Freezing the solution into a container with a cold copper finger at the bottom is called UF, and a honeycomb structure is usually obtained (*39*). Unlike these, centripetal freezing produces a complex but ordered microstructure and imparts an NPR effect to the material. The fabrication schematic of CGM is shown in Fig. 1. In simple terms, the GO aqueous solution is poured into a hollow cylindrical copper mold and sealed with copper, which shows in Fig. 1A. After that, the mold is immersed in liquid nitrogen, followed by freeze-drying and reducing to obtain CGM. In the initial state of freezing (Fig. 1B), a large temperature gradient from the interface between the solution and the copper mold to the center of solution is established; that is a centripetal temperature gradient (*40*). It is known that ice crystals preferentially grow parallel to thermal gradients (*41*). In this case, the ice crystals grow toward the center under the influence of centripetal temperature gradient (Fig. 1B). After the solution is completely frozen, the directional growth of ice crystals has been completed. The ice crystal growth will squeeze the solute to the ice crystal interface and finally forms a 3D skeleton as ice crystal sublimates (Fig. 1D) (*42*–*44*). After that, the GO skeleton was effectively reduced to reduced GO (RGO) aerogel by thermal reduction process (fig. S1).

**Fig. 2.**
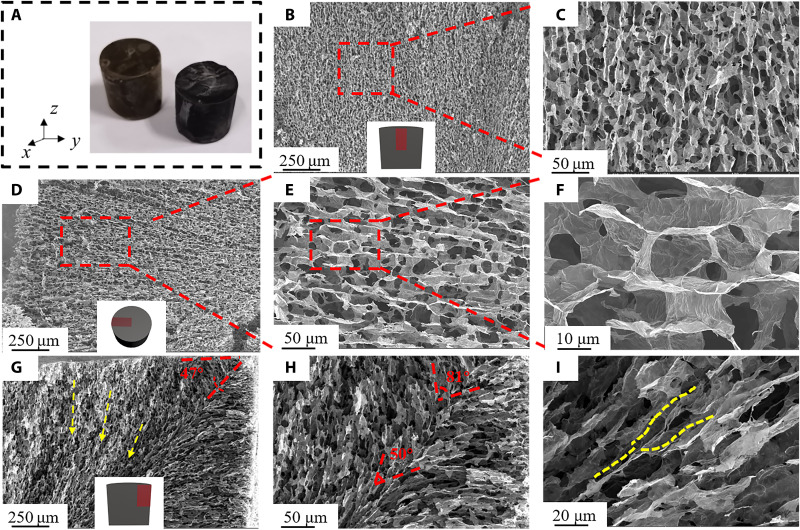
The microstructure of CGM. (**A**) Optical pictures of the sample. (**B** and **C**) Internal morphology of CGM sample in the *z*-*z* direction with different magnifications (250 and 50 μm, respectively). (**D** to **F**) Radial orientation of the material in the *x-y* plane with different magnifications (250, 50, and 10 μm, respectively). (**G**) Diagonal features and specific orientation of channel in the *x-z* plane of the CGM. (**H**) As the channel extends inward, the convergence angle at the diagonal evolves. (**I**) Unique Y-shaped structure on the diagonal.

The microstructure of CGM was captured by scanning electron microscopy (SEM). Figure 2A shows the optical images, where black CGM was obtained. Centripetal freeze-casting gives the graphene scaffold a unique microstructure. Figure 2 (B and C) shows the parallel orientation of the graphene sheets in the *z*-*z* direction, which is similar to the structure formed by UF. The center of the sample in the *z*-*z* direction is weakly affected by the lateral temperature, and the longitudinal temperature gradient has a major effect on growth of the ice crystals. In contrast, as shown in Fig. 2D and fig. S2, in the *x*-*x* (or *y*-*y* direction), the temperature gradient is approximately localized from the edge of the ring to the center, which leads to a radial orientation in the horizontal plane (Fig. 2E). Figure 2F and fig. S2 further illustrate the columnar channels formed by the graphene sheets. The dispersion is freeze-cast from the edge to the center of the mold, and the ice-front velocity is higher because its freezing interface area is reduced during freezing. The higher growth velocity results in narrower pore channels (*38*), as shown in fig. S3. The width of these channels decreased from ~14 μm from near the outside edge to ~10 and 7 μm, in the middle and center, respectively.

The unique microstructure formed by centripetal freeze-casting is reflected in the 47° diagonal (Fig. 2G) that is the *x*-*z*/*y*-*z* direction (owing to the axial symmetry of the cylindrical sample, it exhibits an isotropic NPR in the horizontal plane; only the *x-z* plane is discussed below). There are mutually perpendicular temperature gradients (*x*, *z* directions) in the *x-z* plane, which cause the lamellae to converge on the diagonal. Moreover, the convergence angle becomes smaller on moving closer to the monolith center, as shown in Fig. 2H. As ice crystals draw on, the angle is reduced from 81° to 50°. For example, in the *z* direction, this is caused by the directional growth of ice crystals in the *x-z* plane after disordered nucleation on the surface of the mold. According to the mold design, as the ice crystals in the *z* direction come into contact with the ice crystals in the *x* direction on the diagonal, the former will be affected by the temperature gradient caused by another direction (*x* direction). The double gradient changes the growth direction of the ice crystals. This phenomenon is mutual and brings about the “encounter-compromise” trend, leading to a unique “bouquet-like” structure. Notably, a “Y-type” special structure was observed (Fig. 2I), which is generated when some ice crystals meet during convergence and move together toward the end of the growth process; the end of this growth produces a Y-shaped node.

**Fig. 3.**
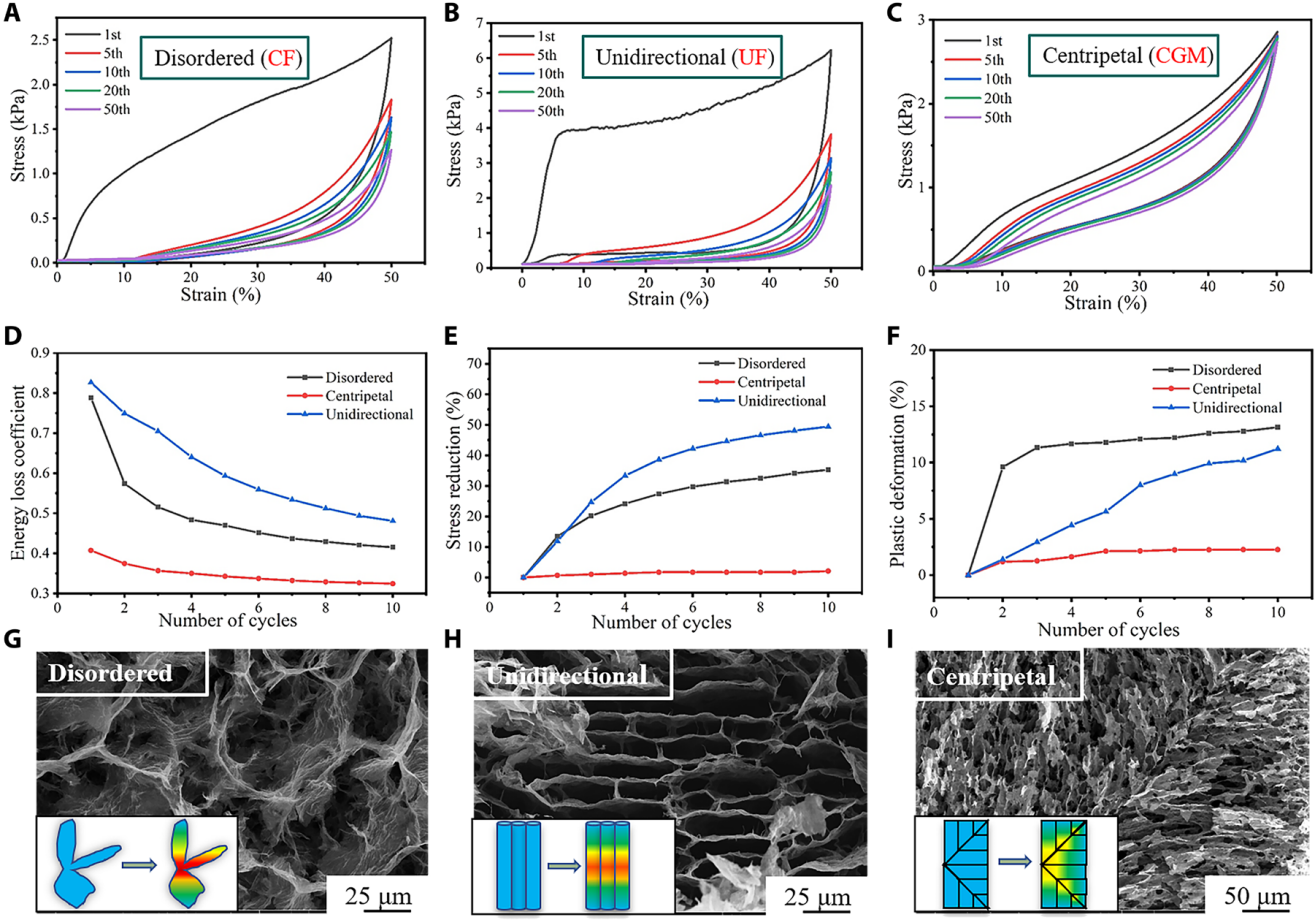
Mechanical performance compared to CF and UF. (**A** to **C**) Stress-strain curves of CF (A), UF (B), and CGM (C) under cyclic compression. (**D** to **F**) Changes in (D) energy loss coefficient, (E) stress reduction, and (F) plastic deformation of disordered, centripetal, and unidirectional structure during the first 10 compression cycles at the maximum strain of 50%. (**G** to **I**) SEM observation of the disordered, centripetal, and unidirectional structure. The inserted models are used to illustrate the force characteristics of the corresponding structure under load. All the samples tested have the same initial solution concentration (8 mg/ml).

To investigate the effects of NPR on the fatigue resistance, CGM was compressed for 50 compression cycles at a maximum strain of 50%, and the data were compared with those of the CF and UF samples. During the deformation process, the maximum stress for CF, UF, and CGM was 2.5, 6.2, and 2.8 kPa, respectively (Fig. 3, A to C). After subjection to 50 compression loops, CF and UF underwent permanent deformations of 16.8 and 12.9%, respectively. CGM only had 4% plastic deformation. UF is more elastic when subjected to axial compression, so transverse fatigue tests were performed. Its maximum load is reduced by 25% from 0.23 to 0.18 N, and the permanent deformation is 12% (fig. S4A). Fibrillar bridges improve UF recoverability. As for CGM, NPR occurs on the end face when it is compressed laterally, but the arc face is expanded, and the stress concentration can easily tear the centripetal channel. Anisotropic structural evolution and stress concentration lead to poor CGM performance (fig. S4B). The relevant parameters for the 1st to 10th cycles were compared to evaluate the mechanical properties of the samples. CF and UF produced large plastic deformation (Fig. 3F), and the maximum stress declined (Fig. 3E) over 10 cycles. The collapse of the inefficient pore structure resulted in a high energy loss coefficient (Fig. 3D), which, thereafter, rapidly decreased. The parameters for CGM became stable after the initial changes, and the structure was slightly damaged.

The performance is often related to the structure. SEM imaging shows that CF often produces random and inefficient structures (Fig. 3G). Such a weak connection causes severe stress concentration at the joint under compression, leading to cell collapse. Moreover, UF led to stacking of the graphene sheets to produce a honeycomb-like structure (Fig. 3H), which effectively improves the mechanical properties of the material, such as the maximum stress. However, this structure is easily fractured at the center of the cell wall as a result of the axial load, and the buckling caused by compression causes fracture at the weak connections. CGM has a radial-like orientation (Fig. 3I), and the existence of lateral channels effectively disperses the load and provides lateral restraint to reduce stress concentration. Notably, the NPR response will cause transverse shrinkage and increase the density during longitudinal compression, resulting in triaxial deformation and even compressive strain in the whole block, allowing the material to bear a higher load and behave more stably. Thus, it is more difficult to produce permanent deformation. On the basis of these results, CGM has better mechanical properties than the counterparts.

**Fig. 4.**
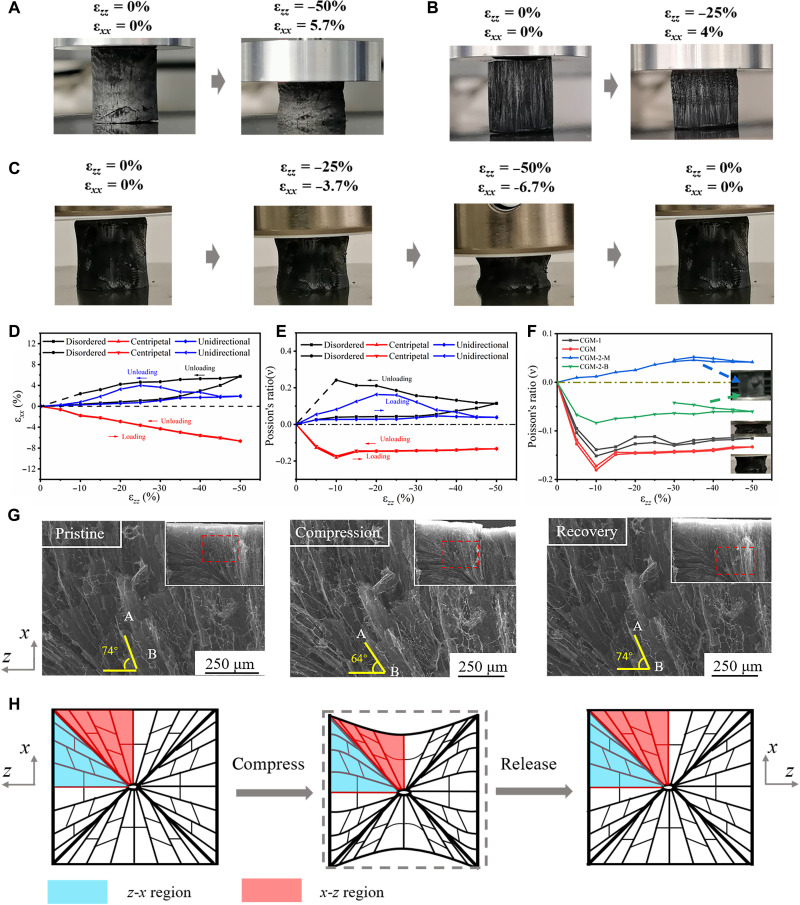
The NPR performance of the as-prepared CF, UF, and radially aligned CGM. (**A** and **B**) Optical images to illustrate positive Poisson’s ratio response of CF (A) and UF (B). (**C**) Sequential optical images showing the NPR response of the radially aligned CGM under compression loading. (**D**) Transverse strain under different longitudinal strain during 50% deformation. (**E**) Evolution of NPR ν as a function of applied ε*_zz_* in a loading-releasing cycle. The dotted line means the plastic deformation part. (**F**) Effects of *h*/*D* on Poisson’s ratio ν. (**G**) Observe microstructure evolution by in situ SEM during compression in the *z*-*z* direction. Inset: A quarter region in the top-right corner. (**H**) Schematic diagram of microstructure evolution during compression.

Centripetal freezing constructs a unique 3D scaffold, and this structure induces an NPR effect when the material is compressed. To quantify the auxetic behavior, the transverse strain (ε*_x_*) and longitudinal strain (ε*_z_*) were measured during the compression process. As mentioned above, Poisson’s ratio, ν, is calculated from the relationship between the transverse and longitudinal strains, namely, ν = − ε*_x_/*ε*_z_*. As shown in Fig. 4C and movie S1, shrinkage occurs along the lateral direction (*x*-*x*/*y*-*y*) when the monolith is compressed in the *z*-*z* direction. This shrinkage appears as a hyperbolic concave pattern in the macroscopic block. As the longitudinal strain increases to 50%, the concavity becomes more pronounced. When the load is released, the degree of lateral contraction is reduced, and the structure gradually returns to the initial state.

Ice crystals produced by different freezing methods undergo orientation and extrusion via diverse mechanisms, and the prepared 3D graphene blocks exhibit different microstructures, which leads to different response patterns in terms of the mechanical deformation. As shown in the optical images (Fig. 4, A and B, and movies S2 and S3), the samples prepared by the conventional freezing method, longitudinal compression produced transverse expansion and the degree of expansion was larger in the unloading process, indicative of a positive Poisson’s ratio. During the longitudinal deformation process, the transverse strain for bulk graphene prepared by the conventional freezing method was consistently positive (Fig. 4D); that is, the Poisson’s ratio was always positive and reached maximum values of 0.24 and 0.16 at 10 and 20% compression, respectively (Fig. 4E). Notably, for CGM, the transverse strain was consistently negative and reached the lowest value of −0.18 at 10% longitudinal deformation (Fig. 4E). This may be attributed to the fact that the microstructure of CF and UF tends to undergo disordered buckling (fig. S5). For CF, it is a 3D honeycomb structure. The cell wall will warp out of plane when subjected to deformation, which undoubtedly increases the lateral dimension of the honeycomb structure, showing a positive Poisson’s ratio, and the stress concentration points tend to collapse as the strain increases. The UF is equivalent to a cylindrical structure, and the lamella expands out of the plane when axially compressed, showing a positive Poisson’s ratio response. Graphene oxide aerogel (GOA) is obtained from the centripetally freeze-casting and freeze drying of GO dispersion, which can transfer to CGM during the following heat treatment process; therefore, GOA and CGM should have similar orientation technically. The similar microstructure makes GOA also exhibit an NPR response during deformation, and the sample shrinks laterally under loading (fig. S6). Unlike GOA, CGM exhibits better elasticity because the π-π attraction between graphene sheets is enhanced after thermal reduction (*39*). The NPR structure and the unique response to the enhancement of the bulk are based on the same material, and there is a huge gap between GO and graphene intrinsic properties that cannot be bridged by simple structural design.

Notably, the Poisson’s ratio of the CGM remained almost constant when the compression was in the range of 20 to 50%. The transverse strain changed steadily with the longitudinal strain. This linear correlation indicates that volume shrinkage occurs in an orderly manner during deformation process, which is beneficial for withstanding higher loads and effectively maintaining structural stability and elasticity under larger compressive strains. During the unloading process, the Poisson’s ratio of CGM followed a trend similar to that during the loading process, which also proves that there was almost no structural damage during the deformation process. Thus, CGM exhibits superelasticity under multiple cyclic loads. The performance of CGM under different strains was explored. Figure 4E and fig. S8A show that CGM maintained good elasticity under the compression over a large range (0 to 50%). The Poisson’s ratio reached the minimum value (approximately −0.18) at 10% compression. The Poisson’s ratio remained consistently negative and gradually stabilized with further deformation. The Poisson’s ratio during unloading was similar to that during loading. The inset shows the optical image of CGM under 0 and 50% strain, respectively, where volume shrinkage was obvious.

It is well known that an NPR is derived from the microstructure of a material. CGMs with different aspect ratios were manufactured to further evaluate the evolution of the microstructure and the NPR effect. As shown in the fig. S7A, blocks with height/diameter (*h*/*D*) = 0.5, 1, and 1.5 were obtained by using different freezing strategies. The curvature at the diagonal was maintained at approximately 45°, and no notable difference was observed. In contrast with the internal radial structure of CGM, the samples with aspect ratios of 0.5 (CGM-1) and 1.5 (CGM-2) showed a special “double Y” orientation (fig. S7, D to F). Although different freezing strategies were used, the driving forces for growth of the ice crystals in the radial and vertical directions during the freezing process were equivalent. Therefore, convergence was terminated early at the double Y node. A low aspect ratio is associated with better stability. The morphologies in the central regions of CGM-1 and CGM-2 were observed. CGM-1 forms an encounter surface in the center, and it is obvious that the graphene sheets facing each other meet and are closely linked, which contributes to its better mechanical behavior (*45*). However, the convergence end point of the graphene sheets in CGM-2 is a line, which is not easy to be tightly combined and not conducive to its recoverability. As shown in fig. S7 (B and C), CGM-1 (*h*/*D* = 0.5) exhibited superelasticity and NPR response. In contrast, CGM-2 (*h*/*D* = 1.5) underwent irreversible deformation. Unexpectedly, opposite Poisson’s ratio responses were detected at the bottom (CGM-2-B) and middle (CGM-2-M) of CGM-2 (Fig. 4F). The internal opposing structural evolutions may induce plastic deformation. It can be determined that the Poisson’s ratio is related to the formation of radial structures.

The NPR effect is attributed to the microstructure design. To elucidate the auxetic behavior, the microstructural evolution of a quarter region in the *x-z* section was characterized by SEM in situ observations during compression. The A-B sheet was selected as the reference point (Fig. 4G), and the angle between the orientation and the *z*-*z* direction was 74°. After compression, the *z*-axis angle decreased to 64°, indicating that the A-B sheet layer shifted diagonally during the compression process. In the corresponding illustration, the material shrinks in the *x*-*x* direction. After the load was removed, the evaluated angle returned to 74°, and the shape was almost unchanged from the initial state. On the basis of this evolution, the mechanism underlying the NPR response was analyzed (Fig. 4H). For the *z*-*x* region, the two ends of the sample in contact with the equipment were fixed because of static friction, and compression caused the internal ordered channels to bend diagonally; for the *x-z* region, the bouquet-like orientation causes one end of the ordered graphene sheet be constrained at the diagonal or central location, and the other end is only weakly constrained. Figure S4 (C and D) shows that almost all graphene channels were gathered at the central location. During compression, the diagonals migrate to the inside, and the ordered graphene sheets undergo tight lamination and rotated close to the diagonals, which results in macroscopic shrinkage in the *x*-*x* direction, leading to an immediate NPR effect. The *z*-*z* zone deformation mechanism also supports the CGM’s ability to withstand large deformation (fig. S8D). It is composed of nearly parallel continuous lamellae in *z*-*z* region. When CGM is deformed, the lamellar is bent, and the deformation location is mainly in the middle of the region. The scaffold did not collapse, and only little debris remained after the load is removed, which is attributed to the fact that the large size of RGO can form a large contact surface to achieve a stronger bond. Compared with other materials, the CGM, which, based on this deflection mechanism, is operative, exhibits a more sensitive NPR response (fig. S8).

The effect of the initial solution concentration on the properties of the metamaterial was explored (figs. S8B and S9). From evaluation of the Poisson’s ratio, all samples show the NPR effect, but there was no obvious NPR effect at lower concentrations (6 mg/ml) and higher concentrations (12 mg/ml) (fig. S9, D to F). From the stress-strain curve and SEM image (fig. S10), it can be concluded that the 3D network is transformed from filamentary connections to layered structures. Moreover, GO has an inhibitory effect on the growth of ice crystals (*34*). Growth of the ice crystals causes the GO layers to form twisted GO fibrils bridging the parallel graphene layers, hindering deflection of the ordered lamellae during deformation, as also proven by the stress-strain curves.

**Fig. 5.**
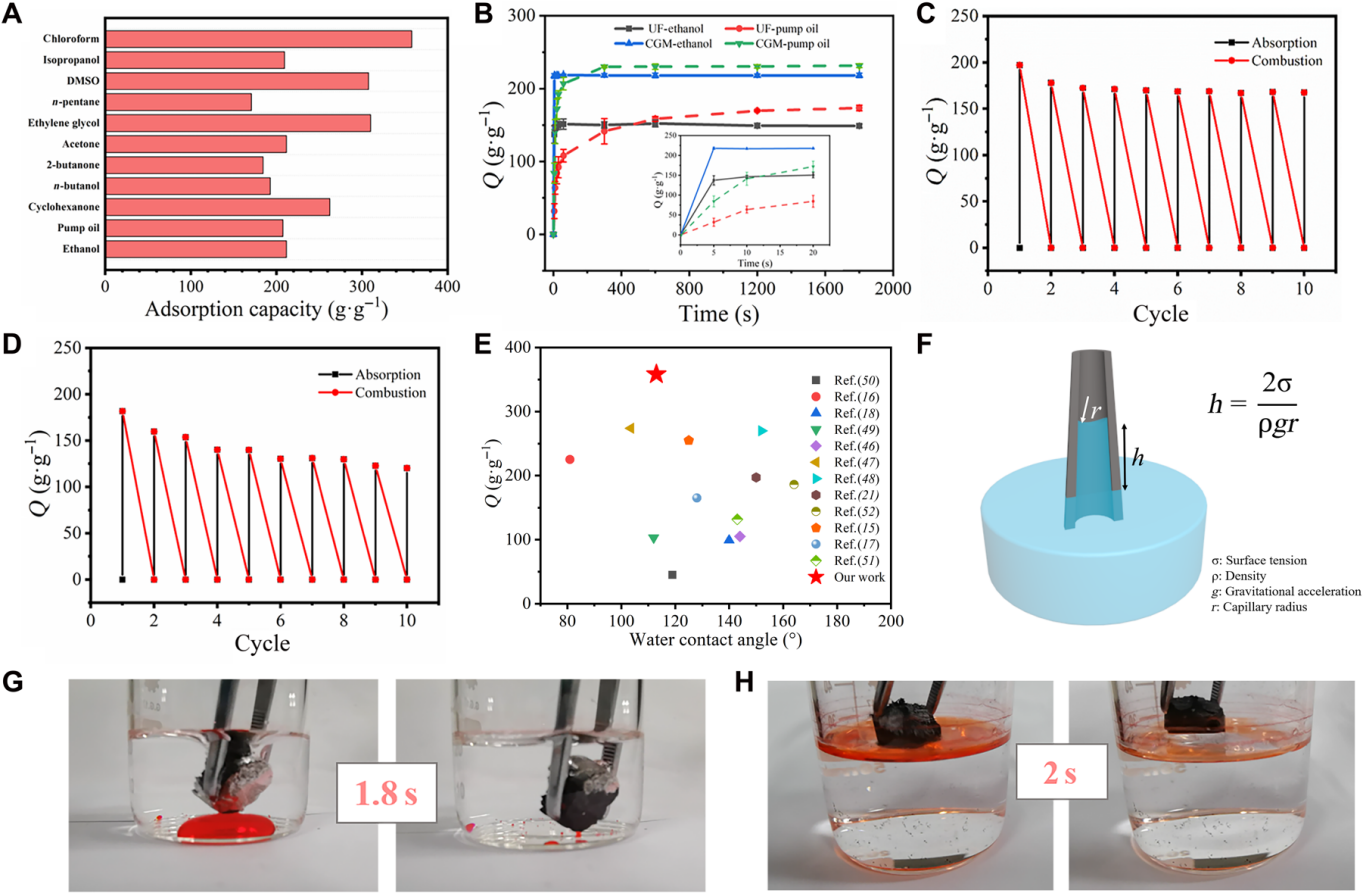
Absorption capacitances of CGM. (**A**) Absorption capacities of CGM aerogel for various organic solvents. DMSO, dimethyl sulfoxide. (**B**) Uptake kinetics of CGM and UF aerogels. (**C**) Cyclic stability of CGM absorption capacity with ethanol adsorption-combustion processes. (**D**) Cyclic stability of UF absorption capacity with ethanol adsorption-combustion processes. (**E**) Chloroform absorption capacities of similar materials for comparison with our work. (**F**) Schematic diagram of capillary tension force. (**G**) Optical photographs of CGM absorbing chloroform (dyed with Sudan III) under water. (**H**) Optical photographs of CGM absorbing *n*-hexane (dyed with Sudan III) on water surface.

CGM can be used as an ideal candidate adsorbent because of its high porosity, large specific-surface area (93.06 m^2^/g), and radially aligned channels; the CGM obtained here was hydrophobic, with a water contact angle of approximately 113° (fig. S11). As shown in Fig. 5A, CGM exhibited excellent adsorption capacity for organic solvents. The capacity for chloroform was up to 358 g·g^−1^. Compared to other similar hydrophobic materials, CGM produced here had a much higher adsorption capability with a low water contact angle (Fig. 5E) (*46*–*52*). Even for high-viscosity liquids, such as pump oil, the adsorption capacity of CGM reached 207 g·g^−1^. This is because the radially aligned channels shorten the transmission path of the adsorbates. The capillary tension provides the driving force for the adsorption process (Fig. 5F). Ethanol and pump oil were used to investigate the kinetics of adsorption on UF and CGM (Fig. 5B). It was confirmed that CGM had a higher adsorption capacity and reached saturation faster. The equilibrium ethanol adsorption capacity of CGM was reached within 5 s. For high-viscosity pump oil, the equilibrium adsorption capacity of CGM was reached in 5 min. Moreover, chloroform (dyed with Sudan III) as a lower layer below water was selectively absorbed by CGM within 1.8 s, as demonstrated in Fig. 5G and movie S5. Correspondingly, *n*-pentane floating on top of the water (dyed by Sudan III) was also rapidly adsorbed by CGM in 2 s (Fig. 5H and movie S6).

The high adsorption capacity is noteworthy. The recyclability of the adsorbent is also indispensable; thus, natural absorption and combustion methods were used to evaluate the recycling stability of CGM. Specifically, CGM and UF were heated in air after fully absorbing ethanol. As shown in Fig. 5 (C and D), during 10 adsorption-combustion cycles, the adsorption capacity of UF was reduced (Fig. 5D), the volume shrank after combustion, and the original shape could not be restored when ethanol was adsorbed again, indicating that the structure was damaged (fig. S13A and movie S7). However, CGM maintained high absorption capacity and reusability. Compared with the precombustion, the pore diameters at different locations all shrink obviously and it leads to the volume shrinkage in the monolith (fig. S13B), which should be related to the capillary force generated by the alcohol transport. As shown in fig. S12, the pore size decreases to 11, 4, and 2 μm from outside to inside, respectively. When the saturated CGM burns, the ethanol in the outer channels is consumed first, and the inner ethanol is continuously transported to the surface because of capillary force. Air cannot enter the CGM because the pores are occupied by ethanol (*53*). Vertical pressure differential provides the same effect as axial load, which allows NPR to work. Therefore, pressure difference is formed between the inside and outside of the CGM, which causes the CGM to contract as the combustion progresses. On the basis of the NPR effect, the lateral active contraction caused by the axial pressure difference can weaken the collapse caused by passive contraction because of the external pressure. We can also observe a slight depression on the sides after burning; thus CGM can undergo greater volume shrinkage during deformation. CGM recovers its original appearance when it adsorbs ethanol again, and the fluctuations are negligible in the subsequent cycles (Fig. 5C, fig. S13, and movie S8).

In summary, GAs with centripetal orientation were prepared by a unique centripetal freeze-casting method. The CGM exhibits an NPR effect (−0.18) and mechanical robustness, derived from the bouquet-like morphology and radially aligned structure. In situ SEM observation confirmed that the macroscopic mold design, combined with deflection of the ordered graphene lamellar, is the major factor leading to the NPR effect. Benefiting from the unique auxetic behavior, CGM shows enhanced mechanical stability and elasticity. The chloroform adsorption capacity is as high as 358 g·g^−1^, the NPR response effectively prevents the force of capillary tension from damaging the microstructure, and CGM exhibits good recoverability. CGM undergoes an increase in the density during deformation, making it potentially applicable in environmental remediation.

## MATERIALS AND METHODS

### Preparation of centripetal freeze-casting device

The centripetal freeze-casting device consists of three parts: a cylindrical hollow copper mold with an inner diameter of 15 mm and an outer diameter of 17 mm and two copper end caps, which were added to form a complete mold (fig. S14). When the interior of the mold is filled with liquid, the atmospheric pressure and surface tension of the liquid ensure that the liquid will not flow out. After placing the device in liquid nitrogen, a centripetal temperature gradient is generated.

### Fabrication of CGM

GO (purchased from Suzhou Tanfeng Graphene Tech Co. Ltd., China) was placed in deionized water, followed by sonication for 30 min to obtain a uniform dispersion (8 mg/ml). The dispersion was poured into the mold and submerged in liquid nitrogen. When the dispersion was completely frozen, a freeze dryer (SCIENTZ-18 N, Ningbo Scientz Biotechnology Co. Ltd., China) was used for freeze drying at 1 Pa for 48 hours, and the CGM was reduced by heating in a vacuum furnace (ZT-80-23 vacuum carbon tube furnace) at 1000°C for 2 hours under argon atmosphere.

The technique used here is the freeze-casting method; therefore, the shape of the CGM is substantially affected by the mold. The size of the cylinder was customized by designing the casting mold, where the main parameters of interest are the diameter and height. The *h*/*D* was designed as 1/1, 1/2, and 3/2, which afforded CGM, CGM-1, and CGM-2 respectively.

### Fabrication of CF

The CF sample was obtained by the common freeze-casting method; that is, the mold filled with the GO dispersion was placed in a refrigerator (−20°C) for 1 day, then freeze-dried, and thermally reduced in the same process.

### Fabrication of UF

The UF sample was obtained by the unidirectional freeze-casting method; that is, a copper cold finger was wrapped by one end of a polyethylene terephthalate film, and the copper cold finger was placed on a copper rod immersed in liquid nitrogen. After the assembly was completely frozen, the same method used for CGM was applied, where the sample was freeze-dried and thermally reduced.

### Characterization

The images of the cross section and longitudinal section of the CGM were captured by a field-emission SEM (FESEM, Hitachi SU8220, Japan), and the x-ray diffraction data for CGM during the thermal reduction process were analyzed using MDI Jade software. Raman spectroscopy was performed using a laser micro-Raman spectrometer (Thermo Nicolet, USA) in the wave number range of 400 to 4000 cm^−1^. The Fourier transform infrared spectrum was acquired in the range of 500 to 4000 cm^−1^ using a Thermo Fisher Scientific iN10 iZ10 instrument. X-ray photoelectron spectroscopy data were recorded using a Thermo Fisher Scientific 250Xi instrument. The compressive strength of CGM was determined by using a universal testing machine (Instron-5566, UK) at a displacement rate of 0.5 mm/min. Digital graphs were captured using a digital microscope (VHX-600).

### Method of calculating Poisson’s ratio

The Poisson’s ratio was calculated by selecting the most obvious position of the transverse contraction under 50% strain, and the transverse strain was determined by calculating the change of the transverse size. A video was recorded during the test of the mechanical properties; video frames were obtained at the same time interval to determine the longitudinal strain (ε*_z_*) and lateral shrinkage (ε*_x_*) of the material using the image editing software ImageJ. Subsequently, Poisson’s ratio was calculated using formula ν = − ε*_x_/*ε*_z_*.

### Oil/solvents adsorption capacity

The oil/solvent adsorption capacity of CGM was evaluated. The CGM samples were weighed and immersed in oil/solvent for an appropriate time. Thereafter, the saturated sample was removed, the excess oil/solvent on the surface was removed using a filter paper, and the samples were weighed again. The absorption capacity (*Q*; g·g^−1^) was calculated as follows

$$Q=\frac{W_{1}-W_{0}}{W_{0}}$$ (1)

where *W*_0_ and *W*_1_ are the weights of the CGM samples before and after the absorption experiment, respectively. All measurements were performed thrice, and the average value is presented as the final result.

### In situ mechanical testing

The CGM was sectioned along the *x-z* plane and clamped it on a jig (the *x-z* plane was kept horizontal) to observe the structure evolution. To prevent the cutter from changing the internal structure during the slicing process, a shallow slit was cut in the CGM with a blade from the side, and then two blades were used to insert the slit to tear the CGM sideways. Then, the prepared sample is clamped on a jig (the *x-z* plane was kept horizontal), and the structure evolution of undamaged region can be observed by FESEM (FEI inspect F50, USA) instrument with a loading rate of 0.5 mm/min.
