# Supplementary material for: Tailoring centripetal metamaterial with superelasticity and negative Poisson’s ratio for organic solvents adsorption
Source: Sci Adv. 2022 Sep 30;8(39):eabo1014. doi: 10.1126/sciadv.abo1014 (PMC9524823; doi:10.1126/sciadv.abo1014)
Supplement: Supplementary file 1 — Figs. S1 to S14 [file sciadv.abo1014_sm.pdf]

Science Advances
AAAS

Supplementary Materials for

Tailoring centripetal metamaterial with superelasticity and negative Poisson's ratio for organic solvents adsorption

Li Tian *et al.*

Corresponding author: Jinshan Yang, jyang@mail.sic.ac.cn; Shaoming Dong, smdong@mail.sic.ac.cn

Sci. Adv. **8**, eabo1014 (2022)
DOI: 10.1126/sciadv.abo1014

The PDF file includes:

Figs. S1 to S14
Legends for movies S1 to S8

Other Supplementary Material for this manuscript includes the following:

Movies S1 to S8

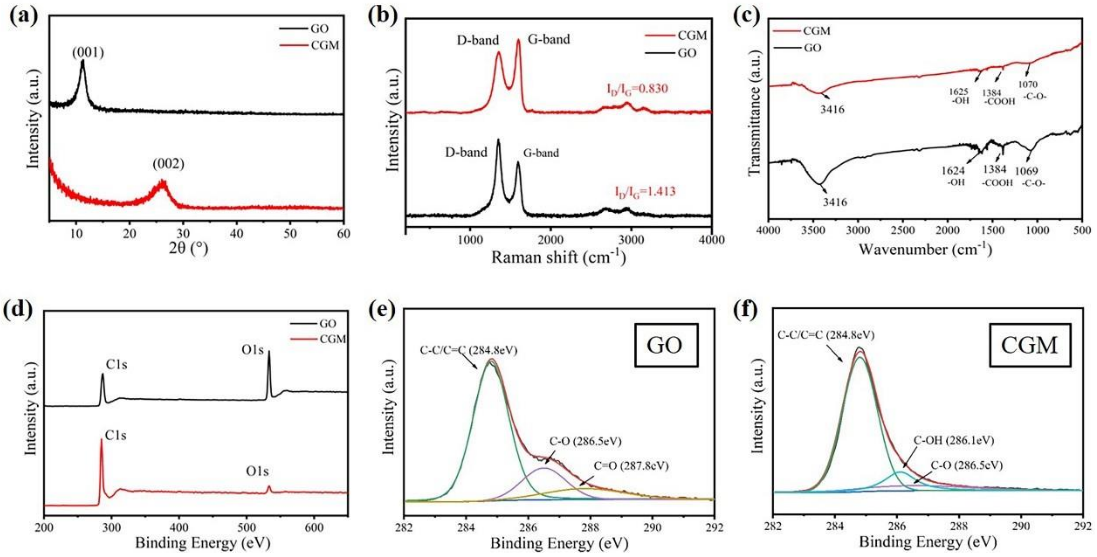

Figure S1. Structural and chemical composition characterizations of GO and CGM. (a-c) XRD patterns、Raman spectra and FT-IR spectrum of CGM. (d) XPS survey spectra for all elements. (e) XPS spectrum for C1s of GO. (f) XPS spectrum for C1s of CGM.

The chemical composition of CGM was investigated. As shown in **Figure S1a**. The XRD patterns of GO and CGM have characteristic peaks of 11.26° and 26.01°, respectively, indicating that the graphene sheets in CGM are highly graphitized and the graphene sheets are effectively stacked through π-π bonds57. Furthermore, D-band at 1351 cm-1 and the G-band at 1594 cm-1 can be clearly observed from the Raman data (**Figure S1b**). The intensity ratio of the D-band and the G-band of GO is ID/IG = 1.413, while the ID/IG = 0.830 of CGM. The decrease in intensity ratio is attributed to the restoration of sp2 domains after thermal reduction. FT-IR and XPS monitor chemical composition during the thermal reduction process. As depicted in **Figure S1c**, After the heat treatment at 1000 °C, the intensity of the peaks was significantly weakened, indicating that the oxygen-containing groups on GO were effectively removed. As demonstrated in **Figure S1d-f**, the XPS spectrum shows that the C/O atomic ratio of CGM is much higher than that of GO, which also proves that GO was effectively reduced to CGM at 1000 °C.

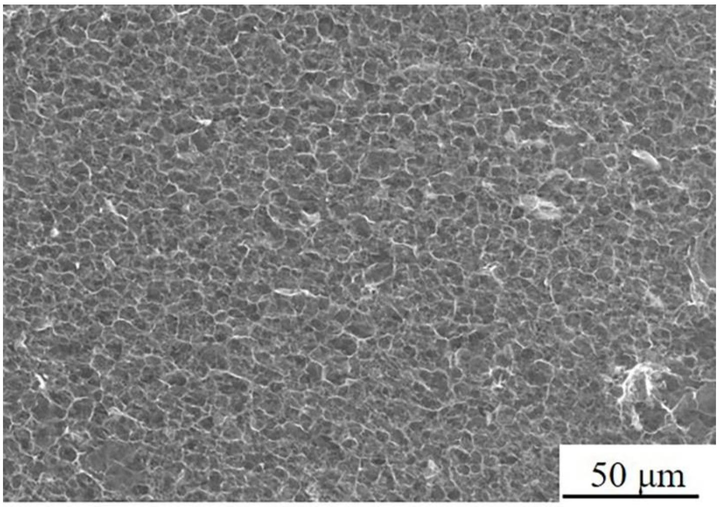

Figure S2. SEM of the graphene channel which perpendicular to the x-y plane.

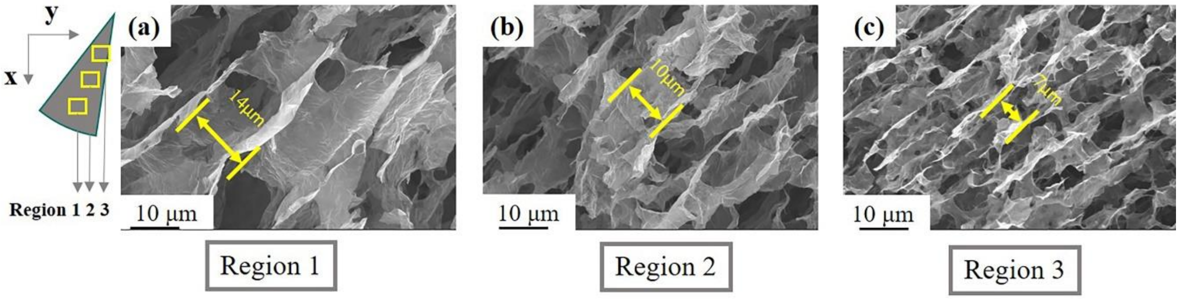

Figure S3. SEM images showing details of the channels in three regions marked.

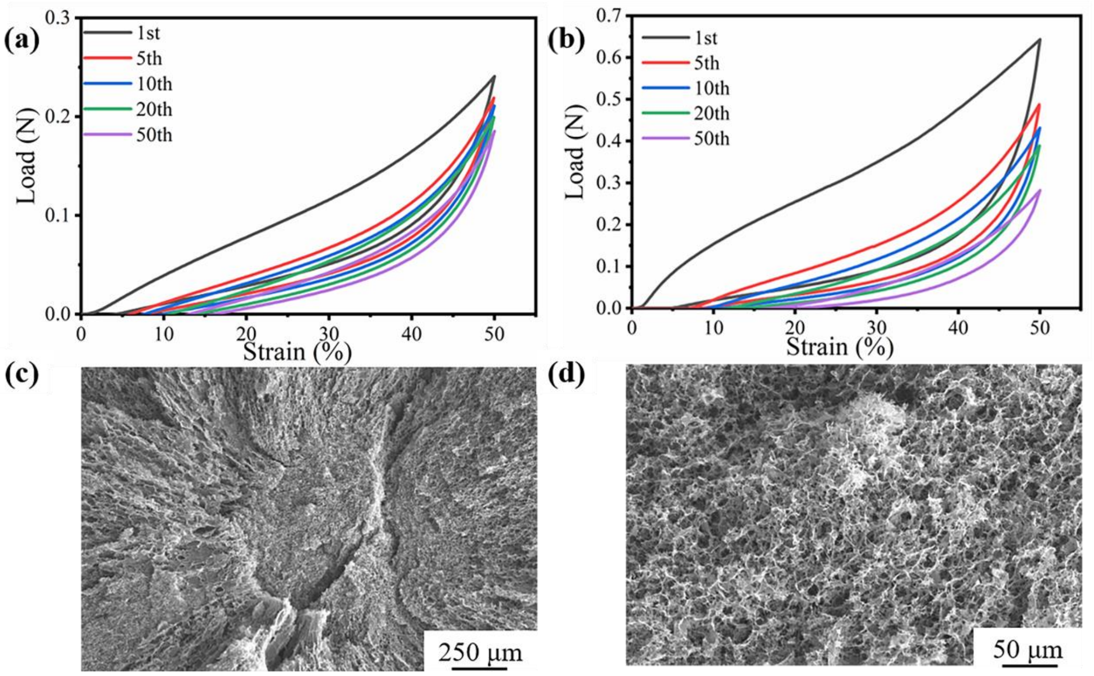

Figure S4. Strain-stress curve of UF and CGM in transverse direction and morphology of CGM in the center. (a) Strain-stress curve of UF. (b) Strain-stress curve of CGM. (c) SEM of the CGM made by centripetal freeze casting which has an ‘approximately radial’ structure; (d) SEM image of the same sample showing the structure at higher magnification.

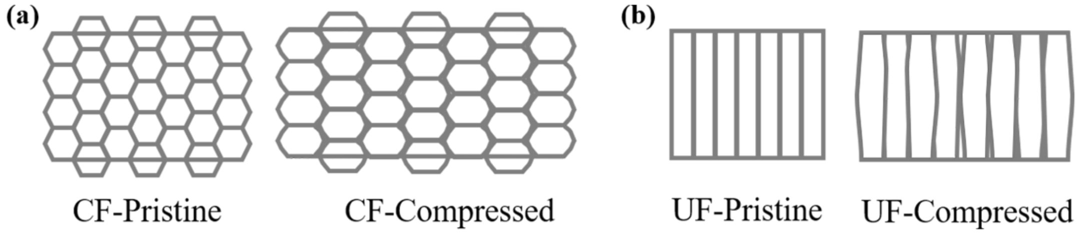

Figure S5. Structural evolution of CF and UF during compression. (a) Structural evolution of CF during compression. (b) Structural evolution of UF during compression.

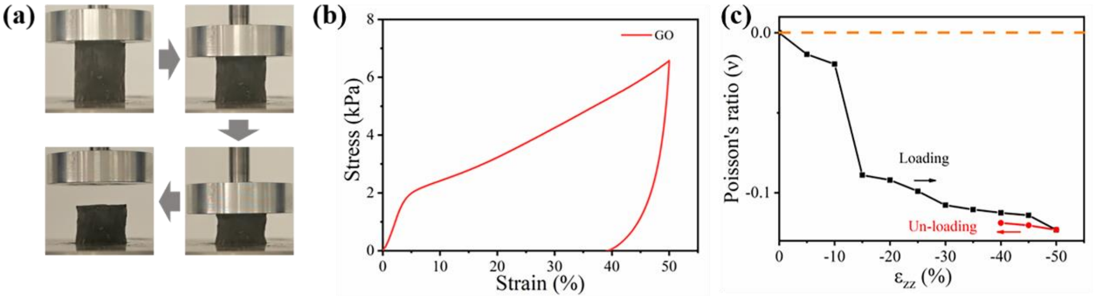

Figure S6. Mechanical properties of GOA. (a) The optical images of GOA during loading cycle. (b-c) Stress-strain curves of GOA. (c) Poisson's ratio performance of GOA.

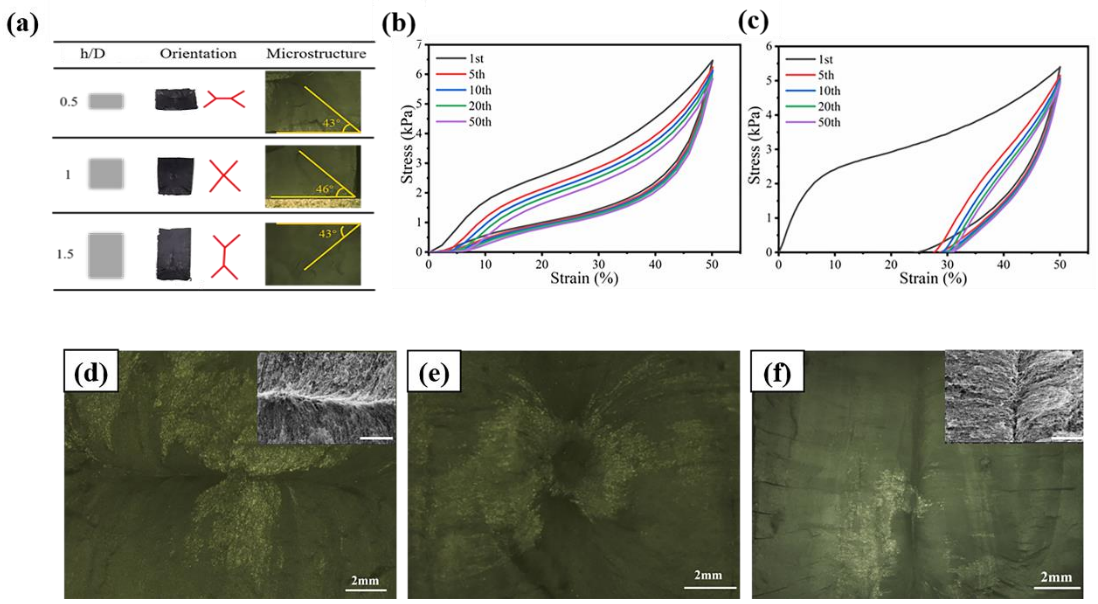

Figure S7. Mechanical properties and morphology **of CGM-1, CGM and CGM-2.** (a) The effects of h/D on Poisson's ratio ν. (b-c) Stress-strain curves of h/D=0.5(b) and h/D=1.5 (c), respectively. (d-e) Digital graphs of CGM-1(d), CGM(e), CGM-2(f). Insets are SEM images, and the scale is 250 μm.

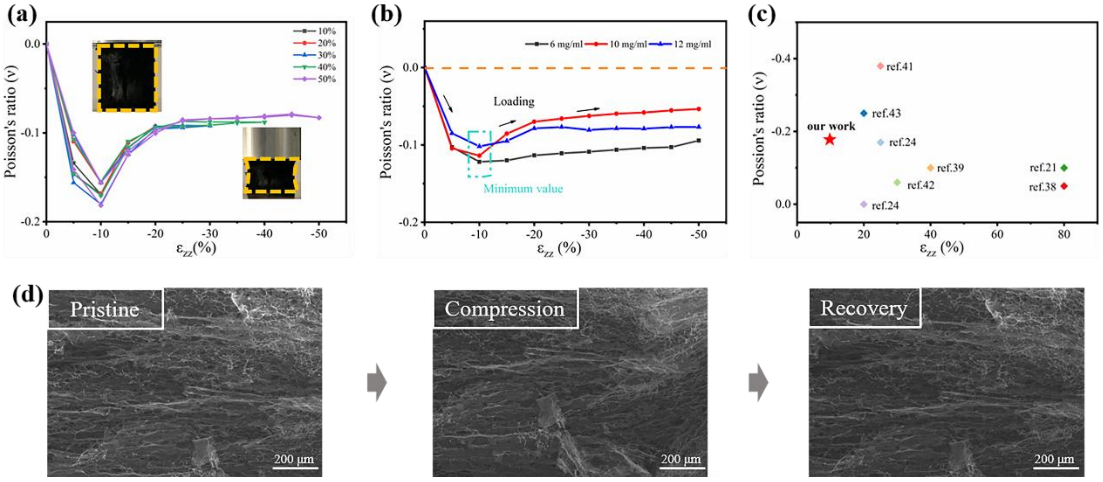

Figure S8. Poisson's ratio of CGM under different conditions and longitudinal structural evolution of CGM. (a) Poisson’s ratio ν of CGM at strains from 10% to 50%. (b) The effects of GO solution concentration on Poisson’s ratio ν. (c) Poisson’s ratio comparison between CGM and other materials. (d) Deformation mechanism in z-z region.

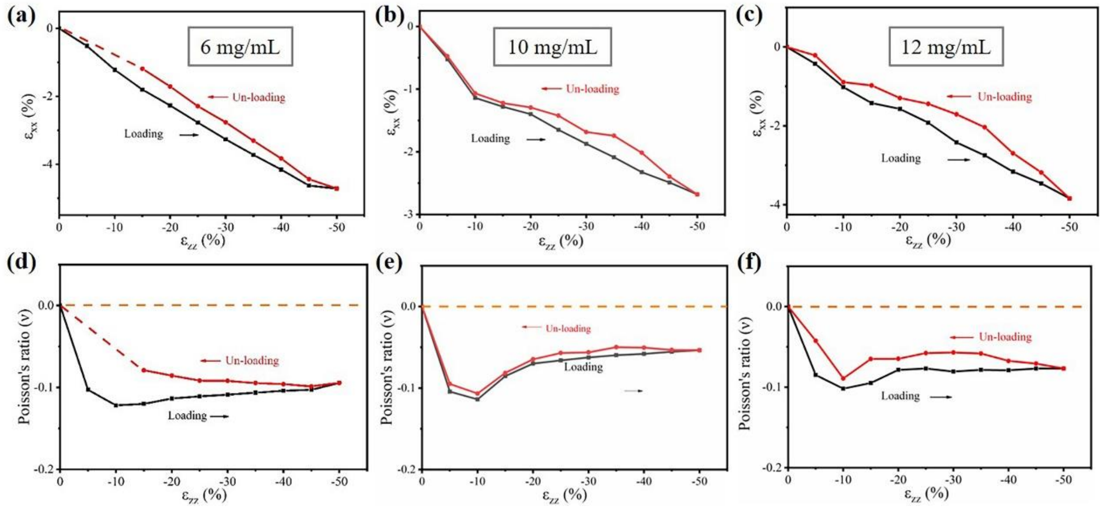

Figure S9. Evolution of transverse strain and negative Poisson's ratio ν under different longitudinal strain during 50% deformation. Initial solution concentration with 6 mg/ml (a, d), 10 mg/ml (b, e) and 12mg/ml (c, f), respectively.

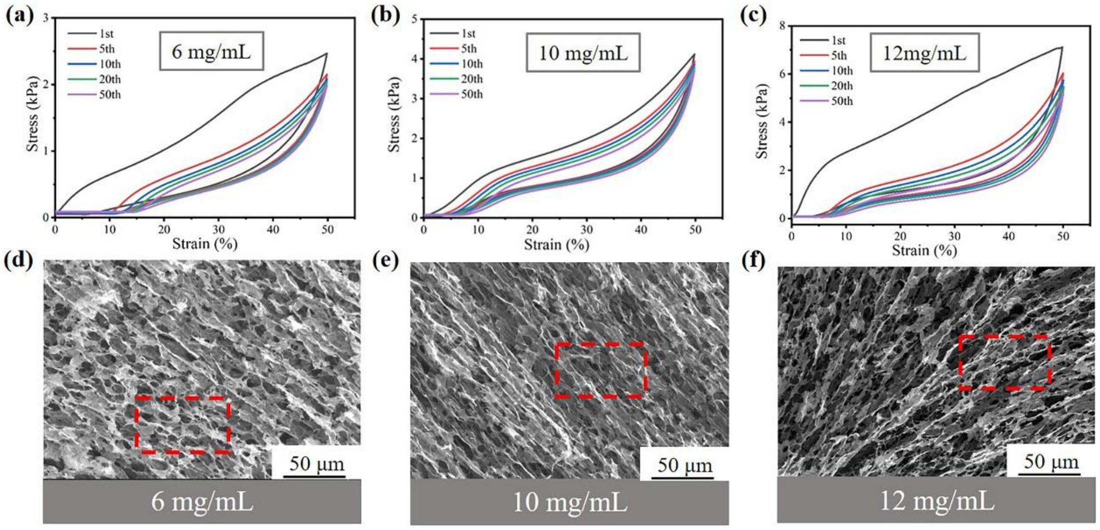

Figure S10. Stress-strain curves and corresponding microstructures of samples prepared under different initial solution concentrations. Initial solution concentration with 6 mg/ml (a, d), 10 mg/ml (b, e) and 12mg/ml (c, f), respectively.

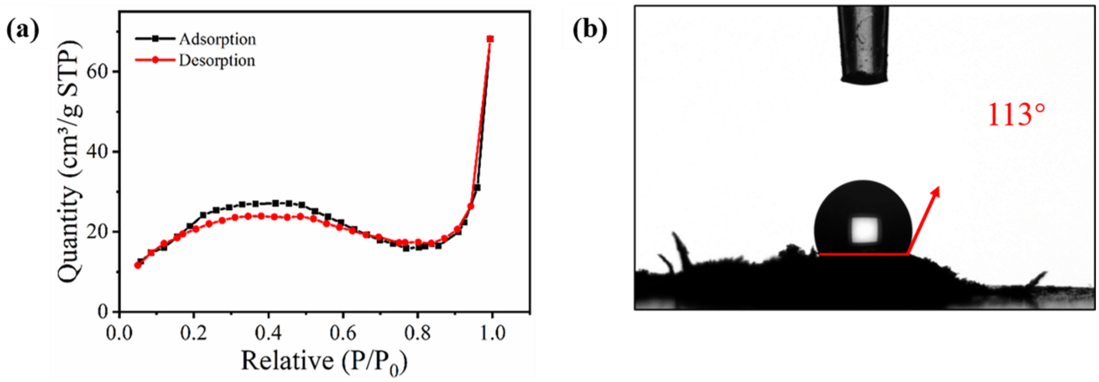

Figure S11. Pore size distribution and wetting angle of CGM. (a) N_2 adsorption/desorption isotherms; (b) Optical images of a water droplet on the surface of CGM, the water contact angle is 113°.

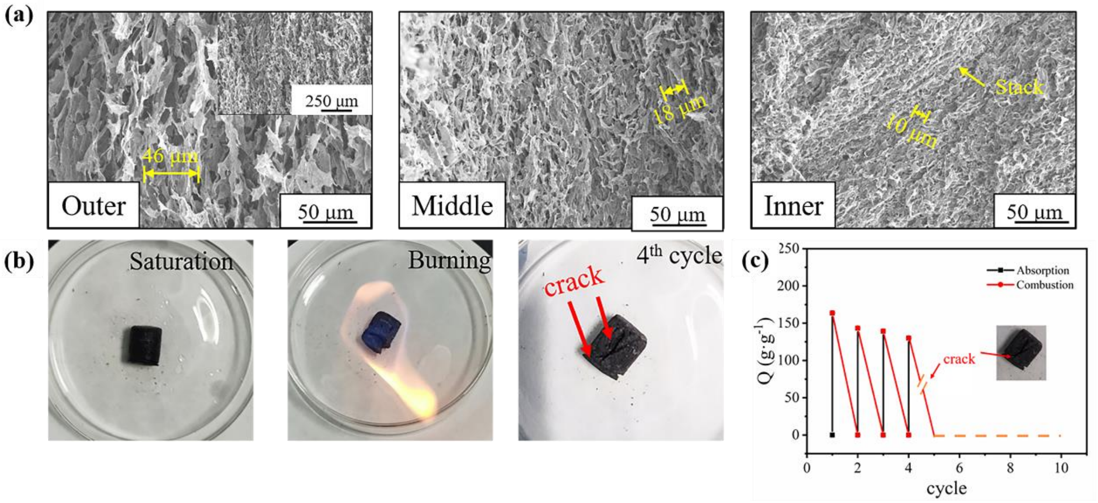

Figure S12. Adsorption capacity of CF. (a) SEM images showing the change in porosity of the radial channels. The inner region exhibits higher density due to greater capillary force generated by narrower channels. (b) CF is broken when subjected to four cycles. (c) The cyclic stability of CGM absorption capacity with ethanol adsorption-combustion processes.

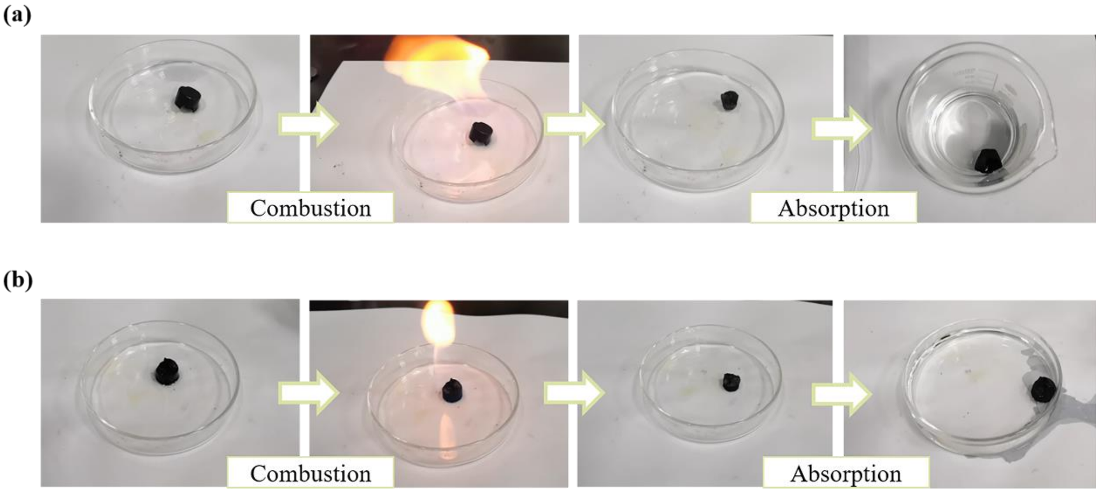

Figure S13. The optical image shows the adsorption-combustion process. After ethanol is completely burned, the volume of CGM (b) and UF (a) decreases, but CGM can restore the original state when it adsorbs ethanol again, while UF cannot.

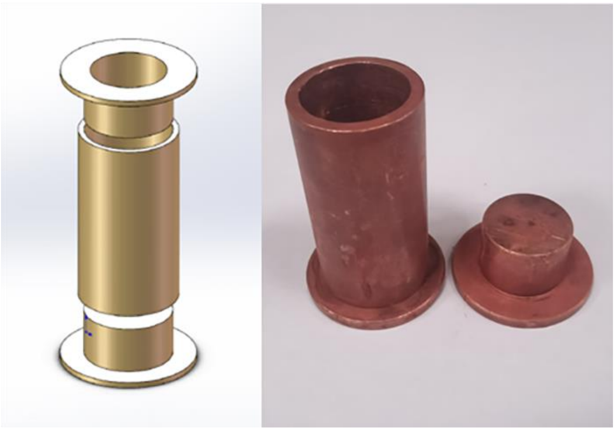

Figure S14. Schematic and optical images of copper mold.

Movie S1.
Shape evolution of CGM during compression

Movie S2.
Shape evolution of UF during compression

Movie S3.
Shape evolution of CF during compression

Movie S4.
Shape evolution of GOA during compression

Movie S5.
Chloroform (dyed with Sudan III) as a lower layer below water was selectively absorbed by CGM

Movie S6.
n-pentane floating on top of water (dyed by Sudan III) was also rapidly adsorbed by CGM

Movie S7.
An adsorption-combustion cycle of UF

Movie S8.
An adsorption-combustion cycle of CGM
